# Intracerebroventricular Coadministration of Protoxin-II and Trace Elements in Rats Enhances the Analgesic Effect of the 1.7 Voltage-Gate Sodium Channel Blocker

**DOI:** 10.1155/2019/8057803

**Published:** 2019-12-28

**Authors:** Gabriela-Dumitrita Stanciu, Andrei Luca, Aurelia Marza, Teodora Alexa-Stratulat, Ionut Tudorancea, Walther Bild, Elena Rezus, Ciprian Rezus, Bogdan I. Tamba

**Affiliations:** ^1^Center for Advanced Research and Development in Experimental Medicine (CEMEX), “Grigore T. Popa” University of Medicine and Pharmacy, 16 Universitatii Street, Iaşi 700115, Romania; ^2^Pneumology Department, “Grigore T. Popa” University of Medicine and Pharmacy, 16 Universitatii Street, Iaşi 700115, Romania; ^3^Department of Physiology, “Grigore T. Popa” University of Medicine and Pharmacy, 16 Universitatii Street, Iaşi 700115, Romania; ^4^Department of Medical Oncology-Radiotherapy, “Grigore T. Popa” University of Medicine and Pharmacy, 16 Universitatii Street, Iaşi 700115, Romania; ^5^Department of Cardiology, “Grigore T. Popa” University of Medicine and Pharmacy, 16 Universitatii Street, Iaşi 700115, Romania; ^6^Department of Rheumatology and Physiotherapy, “Grigore T. Popa” University of Medicine and Pharmacy, 16 Universitatii Street, Iaşi 700115, Romania; ^7^Department of Internal Medicine, “Grigore T. Popa” University of Medicine and Pharmacy, 16 Universitatii Street, Iaşi 700115, Romania

## Abstract

Pain continues to be a global unmet medical need, and the current recommendations for its management require a constant exploration of new drugs that target multiple pain mechanisms, with an improved safety profile and increased treatment adherence. Currently, the enriched distribution and localization within nociceptors of the selective channel blockers and the critical role played by sodium channels in neuronal excitability nominate isoforms as specific targets to generate innovative compounds. In the present report, we verified the hypothesis that coadministration of Protoxin-II, a selective sodium channel inhibitor, and trace elements has direct and improved antinociceptive effects. Groups of seven Wistar rats were treated intracerebroventricularly with a combination of MgCl_2_, CdCl_2_, and ZnCl_2_ and Protoxin-II, respectively, and with Protoxin-II alone (positive) or saline (negative) for controls. Evaluations were performed by nociception assay. Coadministration of these drugs caused an increase in the maximum possible effect of up to 40% as compared with the control groups. Our findings indicate that selective channel blockers continue to be an important nociception target and that the use of trace elements may provide simple but effective means of control over sodium channel blockers' risks, potentially lowering the necessary analgesic doses, thus improving the efficacy and safety profile.

## 1. Introduction

Over the course of the recent decade, pain has become an overwhelming global problem on patients and community, mainly because existing pain therapy exhibits limited efficacy and multiple side effects with potential drug abuse. Worldwide, low back pain represents the main reason for years lived with incapacity counting over 57.5 million patients every year, followed by migraine with almost 45.1 million, the sixth being neck pain with over 28.9 million, while additional musculoskeletal conditions are seventh with more than 28 million patients [1].

Current options for pain management are diverse, including lifestyle changes, physical therapy, counselling, or surgery, and frequently centred on regular pharmacological therapy with opioid or nonopioid drugs, which have not meaningfully transformed in recent years. Therefore, it is a constant need to discover novel molecules that target diverse pain pathways, with an improved safety profile and better treatment adherence. In the past decade, a variety of *in vitro/in vivo* and clinical reports have centred on reviewing the use of common trace elements as analgesics or coanalgesics that increase the medication effects, thus leading to improved pain management or smaller dosage demands [2–5]. Thus, in rodent models, Zn intrathecal delivery caused a significant attenuation of the pain behaviour assessed by the writhing test, while Zn chelators resulted in an important hyperalgesia in the tail-flick test, findings that highlight a direct involvement of spinal Zn in pain modulation [6]. Furthermore, zinc chloride has been shown to have dose-dependent analgesic activity in a rat model of induced neuropathy, even if the form of administration was different (systemic, intraplant, or intrathecal) [7]. Similarly, intraperitoneal zinc chloride administration in a mice study conducted in our laboratory shows an increase in latency of 17% in nociceptive tests and a 25% decrease in pain-associated behaviour in the writhing test [5]. The evaluation through thermonociceptive tests of three diverse doses of magnesium chloride (37.5, 75, and 150 mg/kg) in a murine acute pain model showed direct antinociceptive effects [5]. Moreover, coadministration of Mg and tramadol has revealed an important improvement of tramadol's analgesic activity as measured by the hot-plate and tail-flick tests [4]. Previous studies [4, 5] also revealed a significant analgesic effect for cadmium chloride mostly on visceral pain as assessed with the writhing test.

Significant genetic elements revealed that the voltage-gated sodium channel (Nav) 1.7 has a main impact on pain perception in both humans and mammals [8–10]. Indeed, most Nav1.7 inhibitors presented are only in some measure selective [11], and blockage of sodium channels other than Nav1.7 may exclude the assessment of maximum efficiency of Nav1.7-inhibiting dosages *in vivo*.

Protoxin-II (ProTx-II; *β*/*ω*-theraphotoxin-Tp2a), a potent (IC50 = 0.3 nM) and selective (with >80-fold selectivity than over other subtypes) Nav1.7 blocker [12], was originally isolated from the Peruvian green velvet tarantula (*Thrixopelma pruriens*). The peptide has a mechanism of action vastly optimized via venom development to strongly inhibit ion channels of the nervous system. Pharmacologically, compounds discovered in the past two decades from spider venoms have been recognized as valuable agents for their neuroprotective, antimicrobial, anticancer, and analgesic properties. Current research studies have specified that these toxins may function as antinociceptive agents by acting on ion channels [11, 13].

Therefore, in the current report, we studied whether ProTx-II coupled with common trace elements represents a reasonable target to minimize the nociceptive processing in central pain modulation following intracerebroventricular administration, increase efficacy, and reduce side effects of selective Nav1.7 blocker.

## 2. Materials and Methods

The experimental study was accepted by the “Grigore T. Popa” University of Medicine and Pharmacy Iasi and was performed in accordance with the European legislation, Directive 2010/63/EU, on the protection of animals used for scientific purposes. All protocol procedures have been carried out considering the reduction of animal potential distress and to minimize as much as possible the number of rats for statistical significance. All animals were euthanized at the end of the procedures in accordance with the legislation.

### 2.1. Study Population and Experimental Protocol

A total of 35 healthy young adult male Wistar rats (350–400 grams, 4–6 months old) were used. They were housed in individually ventilated cages (IVCs), containing shaving bedding material, with regular rodent chow and water *ad libitum*. The facility was climate-controlled: 20 ± 4° Celsius, 50 ± 5% relative humidity, and 12-hour light/dark cycles.

All substances were acquired from Sigma-Aldrich GmbH, Steinheim, Germany, and comprised ProTx-II, Mg chloride (MgCl_2_), Cd chloride (CdCl_2_), and Zn chloride (ZnCl_2_). For intracerebroventricular (ICV) delivery of the reagents, a stereotactic system (Stoelting Co, IL, USA) has been used after the rats were anesthetized with ketamine (100 mg/kg) and xylazine (10 mg/kg). Briefly, the head of the rats was mounted in a stereotactic frame and the soft tissues were incised to expose the skull surface and a small hole was drilled for implantation of the guide cannula. The cannula (23 gauge) was placed into the cerebral ventricle following the protocol designed by Sutoo et al. [14]. ICV-cannulated animals were housed in IVCs and allowed to recuperate for 3 days before the experimental procedures were realized. Animals were supervised daily and removed from the study if any neurological deficits were noted, if there was a weight loss greater than >15% over 3 days, or if the cannulas were obstructed.

The behavioural tests, hot plate (HPT), tail flick (TF), and paw withdrawal thresholds (PWTs), have been performed using a protocol presented previously by our group [4, 5]. In the ICV administration, all reagents were freshly diluted in 10 *μ*L of saline. Different groups of 7 rats received one of the following formulations: ProTx-II 0.005 mg/kg for the positive control group, 0.9% saline (10 *μ*l) for the negative control group, ProTx-II 0.005 mg/kg + CdCl_2_ 20 nmol Cd/rat for the Cd group, ProTx-II 0.005 mg/kg + MgCl_2_ 600 nmol Mg/rat for the Mg group, and ProTx-II 0.005 mg/kg + ZnCl_2_ 30 nmol Zn/rat for the Zn group. Behavioural tests were achieved before and after the injection of the substances (15, 30, 45, 60, 75, and 90 minutes). Response latencies were automatically recorded. Behavioural procedures were performed by a researcher blinded to team and treatment. Considering the principles of the 3Rs (Replacement, Reduction, and Refinement) for performing more humane animal research, we selected the doses of Cd, Mg, and Zn after extensive literature study on the use of trace elements as analgesics or coanalgesics [4–7, 14–23], and the selection was refined according to Sutoo and Akiyama [14] to obtain optimal balance between efficacy and safety.

### 2.2. Statistical Methods

The results were analysed using the Prism 8 (GraphPad Software Inc., La Jolla, CA), where two-way ANOVA was used to indicate the presence or absence of statistical differences between groups at the time points taken in the study, followed by Bonferroni post hoc test or just *t*-test, as it was necessary. The groups were checked for consistency. Statistical significance threshold was set at *p* < 0.05. Maximum possible effect (%MPE) was determined using the following formula:

(1) $$\begin{array}{c} \%\text{MPE}=\left[\frac{\text{treatment latency}-\text{baseline latency}}{\text{cutoff latency}-\text{baseline latency}}\right]\times \;100. \end{array}$$

## 3. Results

### 3.1. ProTx-II + CdCl_2_

**Figure 1:**
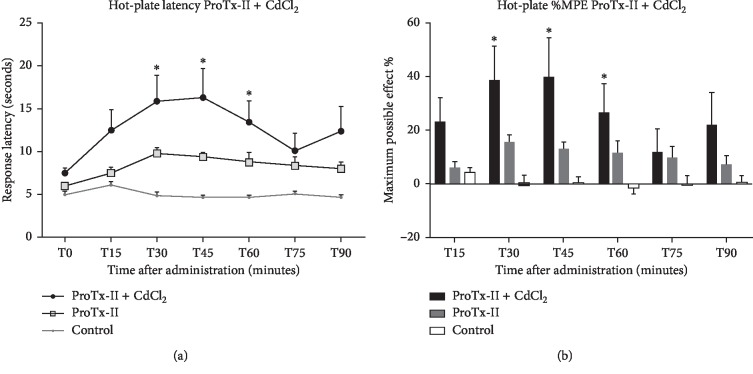
The analgesic effect of Protoxin-II (0.005 mg/kg b.w.) combined with cadmium chloride (20 nmol/rat) in intracerebroventricular administration, compared with Protoxin-II (0.005 mg/kg b.w.) as positive control and saline (NaCl 0.9%, 5 *μ*l/rat) as negative control. A significant difference from the control groups was noted with ^*∗*^ for a *p* value < 0.05. (a) Hot-plate testing with response latency in seconds and evaluated over a period of 90 minutes after administration (represented as mean ± SEM). (b) The maximum possible effect (%MPE) expressed as % value and represented as mean ± SEM.

The TF results revealed that adding CdCl_2_ produces a mild but sustained analgesic effect, not reaching, however, statistical significance (Table 1). The HPT latencies showed a much more coherent effect, with a statistically significant increase in latency starting at 30 minutes for ProTx-II with CdCl_2_ in comparison with vehicle and peaking at 45 minutes (*p* < 0.05) (Figure 1(a)), with the highest MPE value of 40.8% (*p* < 0.05) (Figure 1(b)). Although the effect was sustained 1 hour later (*p* < 0.05), there was a decrease in %MPE to 26.7% (Figure 1(b)).

**Figure 2:**
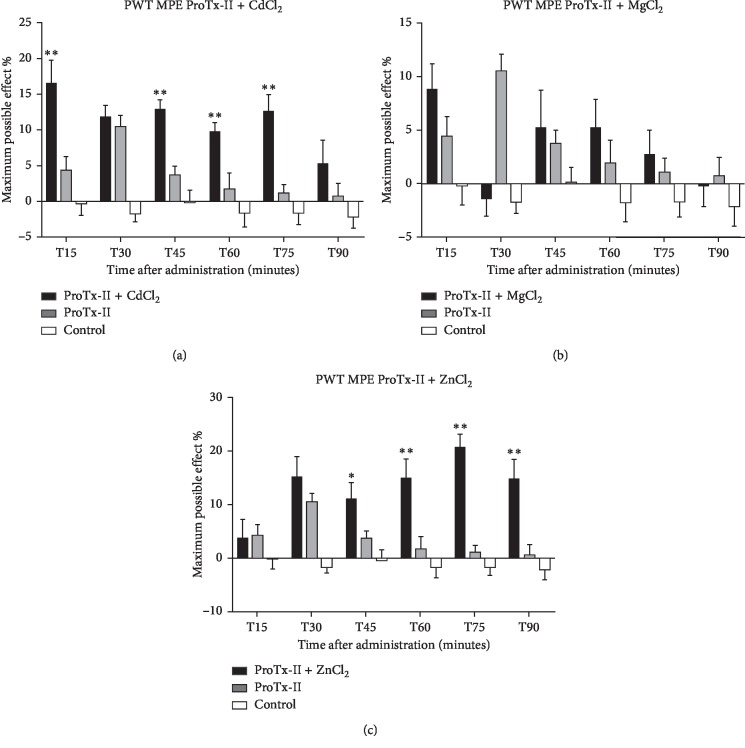
The maximum possible effect over the paw withdrawal threshold test expressed in % and represented as mean ± SEM over a period of 90 minutes. Significant difference from the control groups was noted with ^*∗*^ for a *p* value < 0.05, respectively, and ^*∗∗*^ for a *p* value < 0.01. (a) Protoxin-II and CdCl_2_ with Protoxin-II as positive control and saline as negative control. (b) Protoxin-II and MgCl_2_ with Protoxin-II as positive control and saline as negative control. (c) Protoxin-II and ZnCl_2_ with Protoxin-II as positive control and saline as negative control.

In paw withdrawal thresholds to a noxious mechanical stimulus, average latency and maximum possible effect values of 0.005 mg/kg ProTx-II in combination with 20 nmol Cd in ICV administration produced a sustained analgesic effect that showed a strong statistical significance (*p* < 0.01) (Figure 2(a)). The MPE reached the highest value 30 minutes after administration (16.5%) and maintained significance for a total of 75 minutes. By comparison, the latency in PWT was increased one hour and a half after administration (*p* < 0.05).

### 3.2. ProTx-II + MgCl_2_

**Figure 3:**
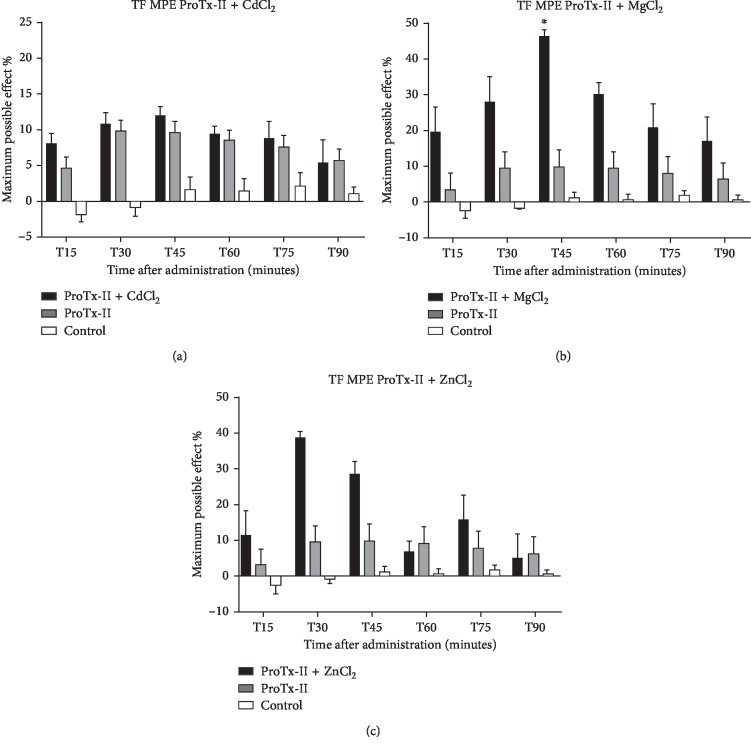
The maximum possible effect over the tail-flick test expressed in % and represented as mean ± SEM over a period of 90 minutes. Significant difference from the control groups was noted with ^*∗*^ for a *p* value < 0.05. (a) Protoxin-II and CdCl_2_ with Protoxin-II as positive control and saline as negative control. (b) Protoxin-II and MgCl_2_ with Protoxin-II as positive control and saline as negative control. (c) Protoxin-II and ZnCl_2_ with Protoxin-II as positive control and saline as negative control.

The TF test showed a sustained antinociceptive effect by adding MgCl_2_. Latency response recorded a more persistent increase in ProTx-II and MgCl_2_ group, with statistical significance even at 60 minutes after ICV administration compared to the positive control group (13.94 seconds as compared to 5.85 seconds in the ProTx-II group (Table 1)). Repeated-measures ANOVA with Bonferroni multiple comparisons showed statistically significant drug effect (F16.40 = 2.734, *p*=0.0028). In contrast to saline control, the groups treated with the MgCl_2_ and ProTx-II combination or ProTx-II alone have shown a significant analgesic effect starting 15 minutes after administration and continued throughout the experiment. The maximum possible effect through combination with the trace element and statistical significance was recorded at 45 minutes (47.45%) while ProTx-II's %MPE in the TF was less than 10% (Figure 3(b)).

**Figure 4:**
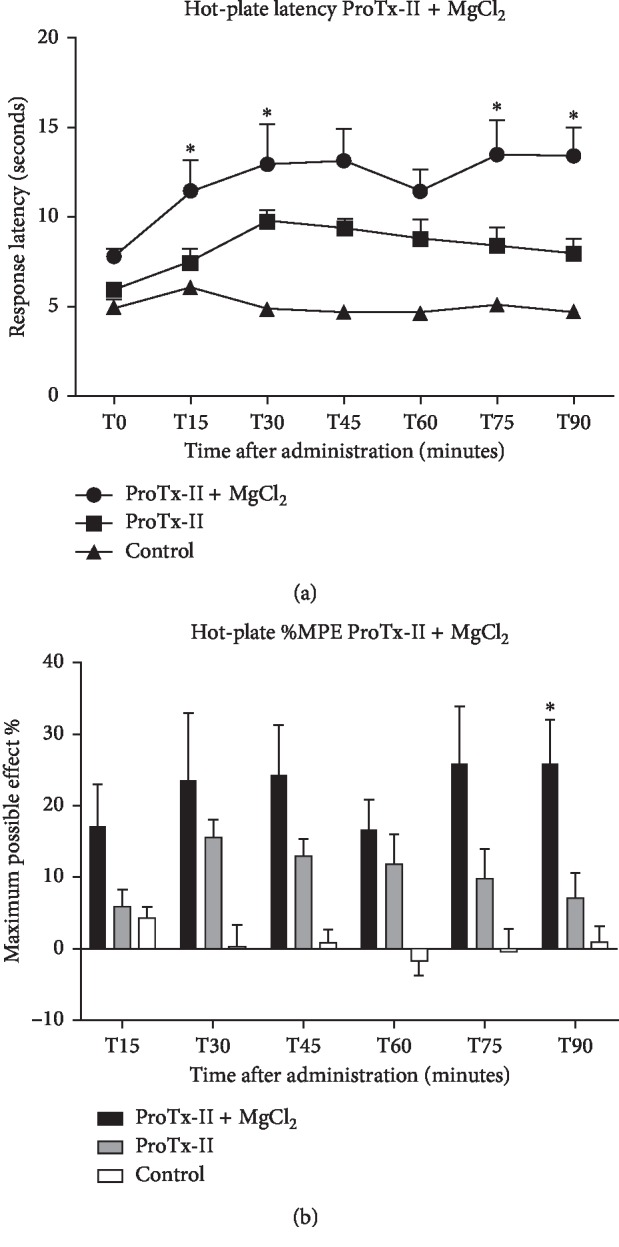
Analgesic effect of magnesium chloride (600 nmol/rat) after ICV delivery combined with Protoxin-II (0.005 mg/kg b.w.) was evaluated in comparison with Protoxin-II (0.005 mg/kg b.w.) as positive control and saline (NaCl 0.9%, 5 *μ*l/rat) as negative control. A significant difference from the control groups was noted with ^*∗*^ for a *p* value < 0.05. (a) Hot-plate testing with response latency in seconds and evaluated over a period of 90 minutes after administration (represented as mean ± SEM). (b) The maximum possible effect (%MPE) expressed as % value and represented as mean ± SEM.

Measurements performed during the HPT show that adding MgCl_2_ enhances continuously ProTx-II's analgesic effect even after 90 minutes, exerting statistical significance (Figure 4(a)). The maximum possible effect calculations of the ProTx-II group were below 15%, whereas the Mg group averages were significant at 90 minutes with a %MPE value of 25.7% (*p*=0.02) (Figure 4(b)).

The PWT test demonstrated an MgCl_2_ combined with Protoxin-II analgesic effect observed at 15 minutes (average latency of 6.21 sec and average MPE of 8.82%) compared with vehicle controls, but no statistical significance was recorded (Figure 2(b)).

### 3.3. ProTx-II + ZnCl_2_

TF test measurements have revealed that ZnCl_2_ leads to improved latency values at 30 minutes after ICV delivery, with *p* < 0.05, which suggests an enhanced analgesic effect, in contrast to the ProTx-II alone. Repeated-measures ANOVA with Bonferroni multiple comparisons has been highlighted a significant substance effect (F12.34 = 1.923, *p*=0.0367). The ProTx-II group average latency responses were 5.97 seconds at 45 minutes and 5.85 seconds at 60 minutes, as compared to Zn group values that were 16.04 seconds at 45 minutes and 15.73 seconds at 60 minutes (Table 1).

Comparing the saline group latency values with those of the group treated with ProTx-II + ZnCl_2_ or ProTx-II alone, statistically significant analgesic effect was obtained on the TF test starting 15 minutes after delivery and persisted throughout the experiment. Average maximum possible effect value was over 35% in the Zn group, while ProTx-II's MPE values in the TF were less than 10% (Figure 3(c)).

**Figure 5:**
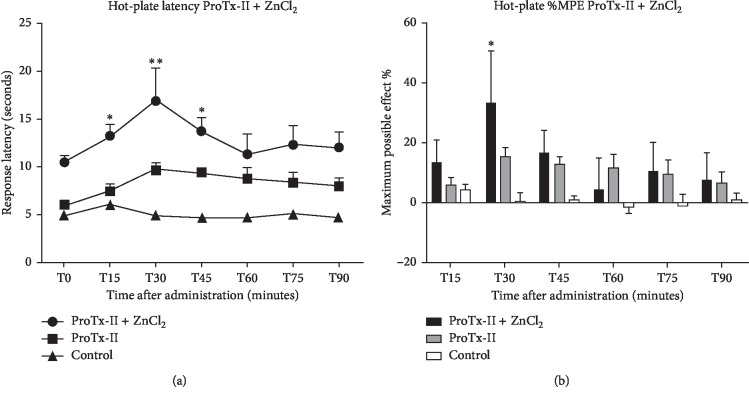
Analgesic effect of Protoxin-II (0.005 mg/kg b.w.) combined with zinc chloride (30 nmol/rat) in intracerebroventricular administration, compared with Protoxin-II (0.005 mg/kg b.w.) as positive control and saline (NaCl 0.9%, 5 *μ*l/rat) as negative control. A significant difference from the control groups was noted with ^*∗*^ for a *p* value < 0.05, respectively, and ^*∗∗*^ for a *p* value < 0.01. (a) Hot-plate testing with response latency in seconds and evaluated over a period of 90 minutes after administration (represented as mean ± SEM). (b) The maximum possible effect (%MPE) expressed as % value and represented as mean ± SEM.

The HPT results revealed that adding ZnCl_2_ causes analogous influence on ProTx-II's analgesic effect with the mention that the effect starts at 15 minutes after administration compared to vehicle, reaching a strong statistical significance at 30 minutes (F7.39 = 15.39, *p* < 0.0003) (Figure 5(a)). By comparison, the MPE effect on the HPT was the highest (33.3%) and significant half an hour after administration (Figure 5(b)).

The PWT test induced a sustained antinociceptive effect by adding ZnCl_2_, starting 45 minutes after delivery maintaining a crescendo effect half an hour later with *p* values < 0.01, compared with vehicle controls. Highest maximum possible effect value was 20.61%, while ProTx-II's maximum MPE values were 10% (Figure 2(c)).

## 4. Discussion

Previous reports have highlighted that ProTx-II may not efficiently cross the sheath of peripheral nerves [15]. Therefore, to confirm the analgesic efficiency but restricted therapeutic influence of ProTx-II in pain modulation, we focused on ICV administration to ensure the Nav1.7 blocker can reach the target sites, knowing that the blood-brain barrier limits the pharmacotherapy of the central nervous system. The above is also sustained by the knowledge that ICV delivery eludes other mechanisms involved in limiting drug distribution at the brain level and facilitates higher drug concentrations in the central compartment.

Several novel compounds have been tested that target Nav1.7 and act as blockers; however, most failed in phase I or II trials, but Nav1.7 is a promising target since successful blocking could alleviate neuropathic pain with acceptable side effects. This is supported by the fact that Nav1.7 sodium channels are encountered in peripheral sensory and sympathetic neurons. The main tests being conducted at this point are comprised of compounds from nonsulphonamide class compounds that target trigeminal neuralgia or postherpetic neuralgia and are in phase II or IIb. Moreover, derived from spider venom, peptide inhibitors of Nav1.7 have been developed that demonstrate high subtype selectivity. The main issue is that important blockers have an intolerable effect which consists of anosmia [24, 25].

Our study suggests that all investigated trace elements improve ProTx-II's antinociceptive effects on nociception after ICV delivery. Coadministration was strongly active in HPT and PWT tests compared with the control groups. Except for the significant antinociceptive effect of ProTx-II and MgCl_2_ on tail flick, differences in effects for coadministration of ProTx-II and trace elements recorded between the hot-plate and tail-flick tests might be attributed to their different mechanism involved in stimuli transmission. The hot-plate test involves higher brain function, being a supraspinal organized response, while tail flick measures a reflexive, spinally mediated response to noxious stimulation. In the absence of neurological dysfunctions following ICV ProTx-II delivery, the insensitivity to pain is like the outstanding phenotype in Nav1.7-null rodent models and humans [16, 17]. In pharmacological reports, the highest tolerated dose of peptide in rats following intrathecal injection was at the dose of 0.1 mg/kg and for intravenous delivery 1.0 mg/kg. Superior doses produced facial lesions or dose-related motor anomalies that evolve from temporary weakness to paralysis of limbs with reducing respiratory frequency and exitus [6, 9]. Remarkably, the presence of lesions to the face has been observed in an experimental Nav1.7 knockout mouse [18], revealing that the process may be sodium channel-mediated rather than peptide-related [19]. In our study, no neurological deficits were noted in the treated rats.

Our findings appear to be in contrast with some earlier reports which highlight the fact that intravenous or intrathecal ProTx-II administration at maximum doses was not correlated with statistically significant inhibition of earlier responses over inflammatory pain [8, 9]. A possible cause for this lack of ability of ProTx-II can be represented by his inability to access the blood-nerve barrier; the theory was sustained by the fact that peptide inhibits the propagation of the action potential of C-fiber just in desheathed nerves [8]. Nevertheless, in their study, Flinspach et al. [11] reported a strong analgesic effect of ProTx-II following intrathecal and perineural administration in rats over nociception tests, by inhibiting Nav from motor neurons.

Interestingly, it has recently come to light that most trace elements (e.g., zinc, copper, and magnesium) might show an analgesic response of their own, in an experimental mouse model of pain [5]. A current report assessed the interaction between some trace elements and diclofenac sodium (tablets and capsules) and revealed that both Zn and Mg amplified the rate of drug release, affecting the pharmaceutical accessibility in the gastrointestinal tract [20]. In our laboratory, coadministration of tramadol and Zn, Mg, or Mn causes a significant improvement of tramadol's results [4].

Based on the corroboration of these hypotheses, associating trace elements with administration of ProTx-II increases the drug's analgesic effect, possibly as a result of voltage-gated sodium channel isoform inhibition by reducing conductance and changing activation toward more positive voltages. A different probability is an additional influence between ProTx-II and MgCl_2_, CdCl_2_, and ZnCl_2_, given that the ProTx-II increased potency over 100-fold for Nav1.7 [10]. Currently, potential association of trace elements with ProTx-II, as analgesics or coanalgesics, is likely to play a crucial role in clinical efficacy and safety control profile. Aiming to reduce the dose of selective sodium channel blockers and thus the potential adverse events associated with long-term administration such as risk of end-organ deterioration, adding a trace element could be a problem solver. However, proper studies must be conducted in long-term administration of these trace elements regarding their own accumulation and separate possible toxic effects.

To our knowledge, the current study is the first report evaluating the influence of trace elements on voltage-gated sodium channel blocker and pain modulation although further studies are required to evaluate whether these trace elements can affect voltage-gated sodium channels, especially Nav1.7, or in the current case affect inhibition of ProTx-II on Nav1.7.

### 4.1. Cadmium Chloride

Even decades after the discovery of this nonessential element toxicity, investigation of Cd as a divalent cation is still an exciting subject in research regarding various physiological roles [16, 21–28].

A normal value of Cd is essential in pain management because this trace element has been shown to influence dopaminergic, glutamate, acid-sensing ion channels (ASICs), and gamma-aminobutyric acid systems. Cd may be a potent neurotoxin even at reduced doses, while Cd deficit can rise oxidative stress and promote mitochondrial abnormalities [24, 25, 27], both being mechanisms vastly implicated in pain modulation [29–31]. Moreover, Cd may inhibit voltage-gated calcium channels and evoked release of neurotransmitters from the nerves, two other potential mechanisms that can stimulate pain transmission [32].

Our results showed that coadministration of CdCl_2_ and ProTx-II exerts an antinociceptive effect, with less significant results in TF test, but with statistically significant effects on the HPT (average MPE of 42.1% at 45 minutes). Our results are similar to earlier studies [4, 5, 33] that showed a moderate action on thermoalgesic testing, with a significant analgesic effect mostly on visceral pain as assessed with the writhing test, looking at the action of Cd on nociception after intraperitoneal administration. It is hard to clarify the analgesic mechanism, though Cd cation is involved in various molecular pathways of pain relief.

However, some reports [12, 34] revealed that CdCl_2_ administration might potently inhibit subtypes of voltage-gated calcium channels. Because Cd and ProTx-II block ion channels by different mechanisms of action, there is the possibility of being a pharmacodynamics interaction between the two compounds.

### 4.2. Magnesium Chloride

A potential role of magnesium supplementation as a coanalgesic in the pain management has become a topic of great interest in recent years. Magnesium, an endogenous inhibitor of N-methyl-D-aspartate (NMDA) receptors and associated ion channels [35], has a confirmed analgesic effectiveness in diverse types of pain in *in vivo* models with diabetic or drug-induced neuropathy [36–38] or ligation of spinal nerve [39]; in humans, Mg has also been successfully tested in a diversity of acute and chronic disorders [36, 38]. The data highlight a direct influence on pain using magnesium with opioid analgesics [40], intravenous general or local anaesthetics [41], and antidepressants [42, 43].

In the current study, adding a dose of Mg to ProTx-II induced different degrees of analgesia in the tests performed, in accordance with the recent data presented by Begon et al. [36] and Tamba et al. [5] even though the routes of administration were different. An animal study looking at the action of magnesium and tramadol coadministration on nociception after intraperitoneal distribution showed that this bivalent cation significantly enhances tramadol's antinociceptive effect in behavioural tests [29]. Considering the drug delivery protocols, our findings suggest that magnesium might exert its effects on pain through selective voltage-gated sodium channel modulation and/or by inhibiting some calcium channels.

Moreover, Mg ions may influence the pain through a number of diverse pathways [44] including the following: (1) decrease in NMDA action, (2) reducing the synthesis of P substance and its action in the dorsal horn, (3) potency of morphine in the presynaptic part of the dorsal horn, (4) a reduction in the action of some calcium channels in the central nervous system, and (5) a reduction in thromboxane A2 or in the synthesis of certain cytokines for the peripheral system [40, 43–50].

### 4.3. Zinc Chloride

Some models of pain have revealed that exogenous application of zinc has an antihyperalgesic effect [20, 47, 51]. Even though the Zn modulation pathways of pain are multiple and complex, it is known to control the functions of many pain-related ions channels, including ASICs [52], voltage-gated Ca^2+^ and Κ^+^ channels [53], and NMDA receptors [48, 51]. Zn deficiency is related to inflammatory processes, phenomenon that plays a crucial role in many pain conditions [54].

We noted a strong antinociceptive effect of zinc in both thermal and mechanical tests. The addition of Zn to ProTx-II augmented MPE by more than 20% as compared to the ProTx-II alone group. Our data may suggest that the additional effect is the result of its own analgesic action and not the effect of ProTx-II metabolism.

Our results are consistent with previous studies that have researched zinc's effects on pain. A study on knockin NMDA mice suggested that zinc has antinociceptive properties by modulating the NMDAs, in which it cannot bind to the NR2A channel [49]. Liu et al. [7] have shown in a rat sciatic lesion model that the zinc chloride has dose-dependent analgesic effects, irrespective of the route of delivery of the compound. Moreover, a recent report on the analgesic effect of intraperitoneal Zn chloride administration shows an increase in TF and HPT latency and a decrease in pain-related behaviour in the PWT [30]. Intrathecal Zn delivery caused an essential reduction of pain behaviour in the writhing test, but Zn chelators persuaded an important hyperalgesia in the TF test, findings that recommend a direct influence of spinal Zn in pain modulation. Zn in the central nervous system modulates glutamatergic transmission and antagonizes NMDAs [55]. Both *in vitro* and *in vivo* studies have exposed that zinc controls glycine, adenosine triphosphate, and g-aminobutyric acid receptors [7, 8] and impacts pain through the ion channels [10]. Given zinc's variety of actions in the nociceptive pathway, it is possible that a small change in free extracellular zinc concentration could have a large effect on nociceptive transmission.

Constant research focused on the identification of trace elements for association with selective sodium channel blockers might provide positive results and could become new therapeutic sources in pain management.

## 5. Conclusions

Our findings in a rodent model indicate that the use of trace elements may provide means of control over Nav1.7 channel blocker side effects, with improved efficacy and increased safety profile. Future research and therapeutic perspectives with respect to pain management should address multiple dose-effect studies of Nav 1.7 channel blockers alone or in combination with analgesic or coanalgesic agents for the development of novel therapeutics against the pathophysiological states of pain.

## Figures and Tables

**Table 1:** Average latency values for CdCl_2_, MgCl_2_, or ZnCl_2_ and ProTx-II coadministration assessed by the hot-plate and tail-flick tests.

Drug	0 minutes	15 minutes (%)	30 minutes (%)	45 minutes (%)	60 minutes (%)	75 minutes (%)	90 minutes (%)
ProTx-II HPT	6.04	7.47	9.79	9.4	8.83	8.37	7.97
ProTx-II TF	5.65	5.70	6.01	5.97	5.85	5.38	4.97

ProTx-II + CdCl_2_ HPT	7.52	12.48	15.93	16.28	13.37	10.12	12.4
ProTx-II + CdCl_2_ TF	6.03	6.34	6.43	6.51	5.82	5.75	5.68

ProTx-II + MgCl_2_HPT	5.88	6.48	9.33	9.28	8.52	8.98	11.47
ProTx-II + MgCl_2_ TF	9.23	9.89	10.25	11.95	13.94	10.21	9.87

ProTx-II + ZnCl_2_ HPT	10.2	13.24	16.56	13.98	11.12	12.02	11.86
ProTx-II + ZnCl_2_ TF	8.63	11.01	12.96	16.04	15.73	14.78	14.56

Saline HPT	5.03	6.12	4.93	4.73	4.65	5.12	4.68
Saline TF	−1.69	−0.58	2.35	2.12	1.67	3.12	1.13

## Data Availability

The statistical data used to support the findings of this study are included within the article.

## Abbreviations

Mg: Magnesium
MgCl_2_: Magnesium chloride
Cd: Cadmium
CdCl_2_: Cadmium chloride
Zn: Zinc
ZnCl_2_: Zinc chloride
Nav: Voltage-gated sodium channels
ProTx-II: Protoxin-II
ICV: Intracerebroventricular
IVCs: Individually ventilated cages
HPT: Hot-plate test
TF: Tail-flick test
PWT: Paw withdrawal threshold test
NMDA: N-Methyl-D-aspartate
ASICs: Acid-sensing ion channels
Voltage-gated Ca^2+^ and Κ^+^ channels: Voltage-gated calcium and potassium channels.
